# The Effect of Post-Spinal Hypotension on Cerebral Oxygenation Using Near Infrared Spectroscopy and Neonatal Outcomes in Full Term Parturients Undergoing Lower Segment Caesarean Section

**DOI:** 10.7759/cureus.90770

**Published:** 2025-08-22

**Authors:** Krithikabrindha Velusamy, Amit Kumar, Shailendra Kumar, Sana Y Hussain, Puneet Khanna, Nishant Patel

**Affiliations:** 1 Anesthesiology, All India Institute of Medical Sciences, Delhi, IND; 2 Anesthesiology, All India Institute of Medical Sciences, New Delhi, IND

**Keywords:** caesarean section, cerebral oxygenation, hypotension, neonatal outcomes, nirs, post spinal hypotension, spinal anaesthesia, vasopressors

## Abstract

Background

Cerebral autoregulation is a homeostatic process that maintains constant cerebral blood flow during hypotension produced by spinal anesthesia. Beyond the auto-regulation range of mean blood pressure (MBP), the brain is vulnerable to ischemia and hyperperfusion-induced cerebral edema when blood pressure is above the auto-regulatory threshold. Spinal hypotension may cause a fall in regional cerebral oxygenation (C-rSO₂) below the lower limit of autoregulation in some patients. In this study, we focused on finding the relationship between C-rSO₂ changes measured by near infrared spectroscopy (NIRS) and the decrease in MBP following spinal anesthesia. We also measured the impact of spinal hypotension on neonatal Apgar scores, acid-base variations in neonates, and postoperative delirium in parturients.

Methods

This was a prospective, observational study. Eighty-six parturients undergoing lower segment caesarean section were monitored using NIRS for cerebral oxygenation continuously, and MBP was recorded every minute preoperatively and up to 30 minutes post-induction of spinal anesthesia. Neonatal Apgar scores were noted at the first and fifth minutes, and umbilical cord blood analysis was done. Cognitive function and the possible presence of delirium were evaluated on postoperative day (POD) one and two.

Results

The fall in C-rSO₂ (27.77 ± 11.54%) did not correlate with the fall in MBP (40.33 ± 9.7%). The highest fall in C-rso2 (4.65 minutes) preceded the maximal fall in MBP (5.68 minutes). MBP was maintained within 20% of the preoperative value using vasopressors. Ephedrine was more effective than phenylephrine in increasing C-rSO₂ (p<0.05). No significant changes were observed in neonatal Apgar or acid-base status. No postoperative delirium was observed in parturients who had hypotension during the procedure.

Conclusion

Although no significant correlation was found between MBP and C-rSO₂, NIRS effectively predicts post-spinal hypotension earlier than intermittent non-invasive blood pressure (NIBP) monitoring. There were no adverse maternal and neonatal outcomes if the hypotension was corrected. Ephedrine was found to be superior to phenylephrine in improving cerebral oxygenation, whereas both had a similar impact on neonatal outcomes.

## Introduction

Spinal anesthesia is the preferred method for cesarean deliveries due to its reduced risk profile compared to general anesthesia. However, post-spinal hypotension affects 70-80% of parturients undergoing cesarean section [1]. Hypotension causes cerebral hypoperfusion and subsequent reduced oxygen tension in brain tissue, which may lead to maternal postoperative delirium and uteroplacental hypoperfusion [2], leading to adverse neonatal outcomes like hypoxia and acidosis [3,4].

Near-infrared spectroscopy (NIRS) is a non-invasive method used to measure cerebral oxygenation by assessing oxygenated and deoxygenated hemoglobin concentration in the brain using the near-infrared portion of the electromagnetic spectrum. While NIRS has been widely used in cardiac surgeries, its application in obstetric anesthesia is limited. Some studies found a fall in blood pressure correlates with a fall in cerebral regional oxygen saturation (C-rSO₂) [5], whereas other studies found no correlation [6]. Cerebral autoregulation maintains the cerebral blood flow constant when the mean blood pressure (MBP) remains between 60 and 150 mmHg. We hypothesize that hypotension after induction of spinal anesthesia might correlate with cerebral oxygen saturation and may influence the neonatal outcomes, like Apgar scores and acid-base status. Accordingly, the effect of post-spinal hypotension on cerebral oxygenation and its correlation with maternal hypotension were studied. We also planned to study the correlation between the cerebral desaturation events and maternal postoperative delirium.

## Materials and methods

This prospective observational study was conducted at the Department of Anesthesiology, Pain Medicine, and Critical Care in a tertiary care apex medical center. Following Institutional Ethics Committee approval (IECPG-496/30.06.2022), the study was registered in the clinical trial registry of India (CTRI/2022/08/044864). The sample size was based on a previous study by Fassoulaki et al. [7] with an expected 10-unit decrease in C-rSO₂, 90% power, and α=0.05; an estimated sample size of 44 was calculated. To account for dropouts, the target sample size was increased to 60.

**Figure 1:**
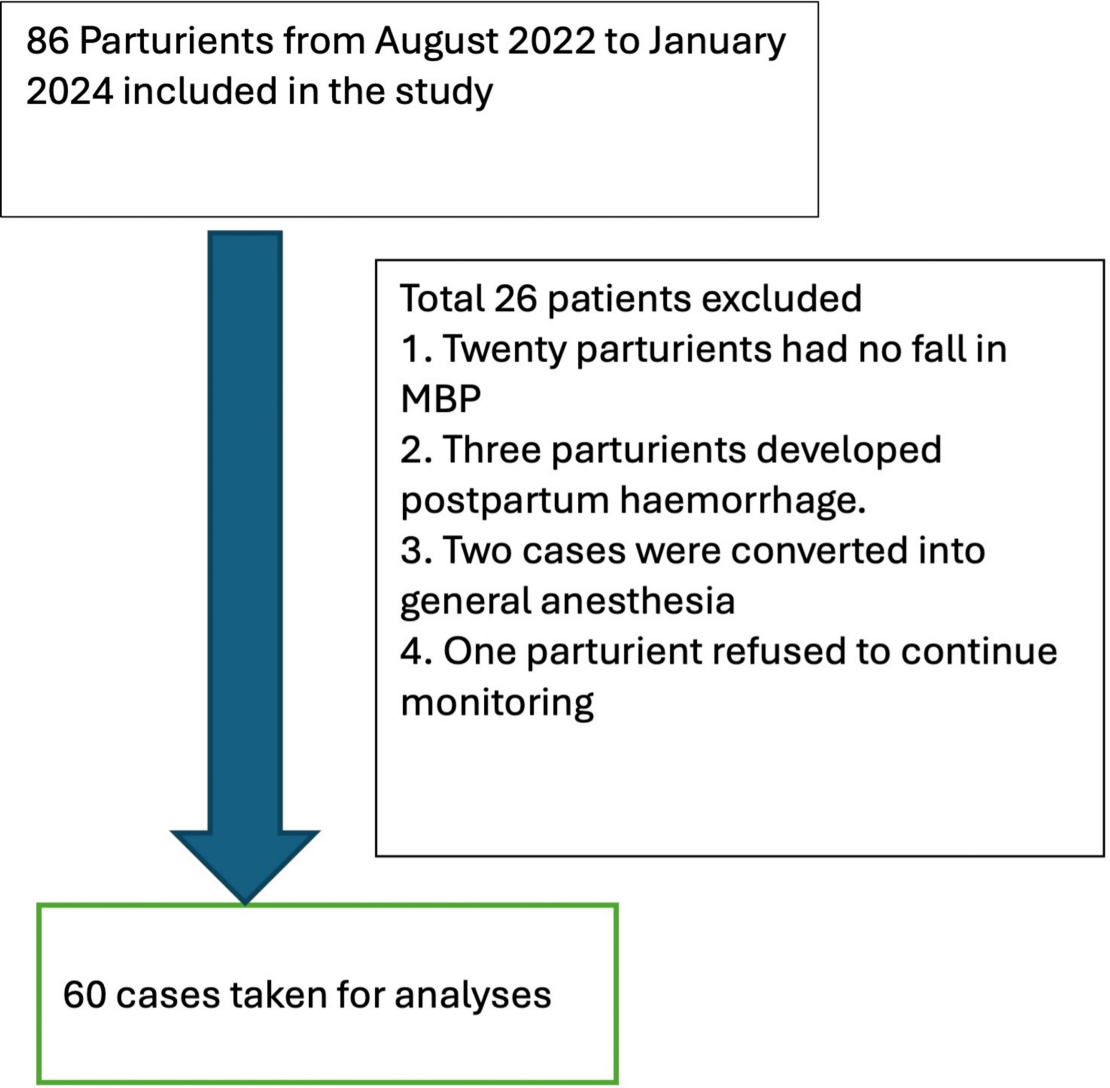
Strobe diagram MBP - mean blood pressure

Eighty-six women of twenty to forty years of age undergoing lower segment caesarean section (LSCS) (sixty-four elective and twenty-two emergency cases) with an American Society of Anesthesiologists (ASA) score of two were included in the study. Parturients with preeclampsia, abnormal level two antenatal scans, antepartum and postpartum hemorrhage were excluded from the study. For analysis, three patients who developed postpartum hemorrhage, two patients who needed to be converted into general anesthesia, and the patients who did not develop hypotension were excluded from analysis. A total of 60 patients were considered for final analysis. The strobe diagram for the study is shown in Figure 1. 

Patients who fit the criteria and who were willing to take part in the study were included. Informed written consent was taken, and the patient information sheet was provided to all patients. Routine investigations, including complete blood count, hematocrit, and platelet count, were noted. All the patients were asked about their fasting status. They were found to be more than eight hours of fasting for solid food and more than two hours for clear fluids in elective cesarean sections. Out of 22 emergency cesarean sections, only four were inadequately fasting; the rest were all adequately fasting. Informed written high-risk consent for aspiration was obtained from the parturient who had inadequate fasting. Anti-aspiration prophylaxis with injection metoclopramide 10 mg and injection ranitidine 50 mg was given to all parturients irrespective of fasting status. ASA standard monitors such as pulse oximeter, NIBP, and electrocardiography (ECG) were attached. Baseline values of pulse rate, SpO2, blood pressure, and ECG were recorded. The NIRS sensor (INVOS oximeter; Medtronic, Dublin, Ireland) was attached to the forehead of the patient just below the hairline, and its baseline readings of cerebral oxygenation on the right and left frontal regions were noted. After securing an intravenous cannula, 500 ml of isotonic crystalloid solution was administered to all the patients and given over 10-15 minutes during the subarachnoid block. Subarachnoid block (SAB) was performed in a sitting position at the level of L4-L5 in all the patients using 0.5% hyperbaric bupivacaine (H) (9-10 mg). With fentanyl 20 mcg as an adjuvant, the individual anesthesiologist determined the dose as per institutional protocol. Immediately after SAB, the patient was returned to a supine posture. Left arm mean blood pressure (MBP) readings were noted at every one-minute interval using noninvasive blood pressure monitoring (NIBP) until thirty minutes after subarachnoid block. If any patient experienced any discomfort or pain in the arm in which the NIBP was attached, it was shifted to the other arm. All the patients had comparable blood pressure readings as measured pre-procedure to exclude subclavian artery stenosis, which could distort blood pressure in the arms.

**Figure 2:**
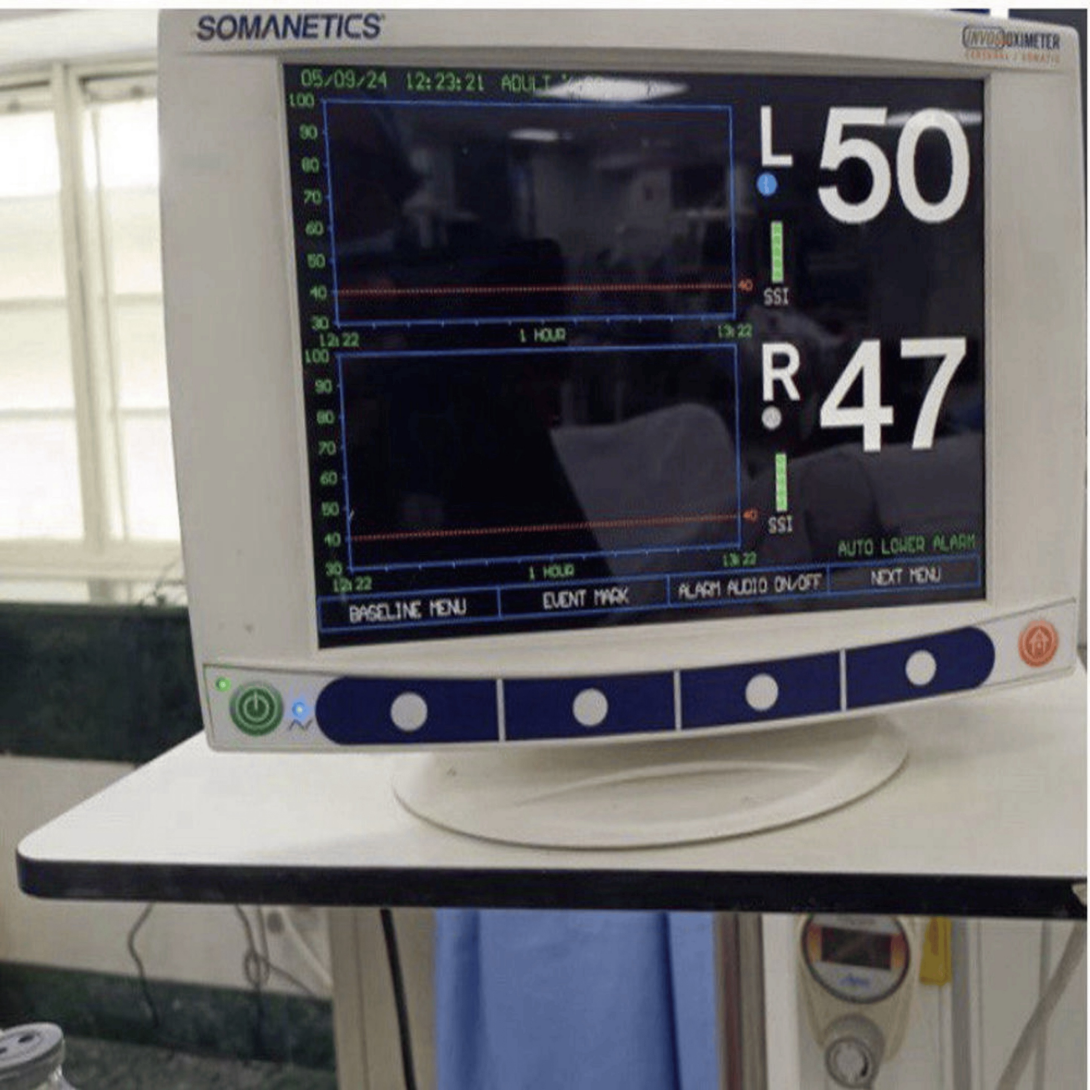
NIRS monitor showing rSO2 of right and left side NIRS - near infrared spectroscopy

**Figure 3:**
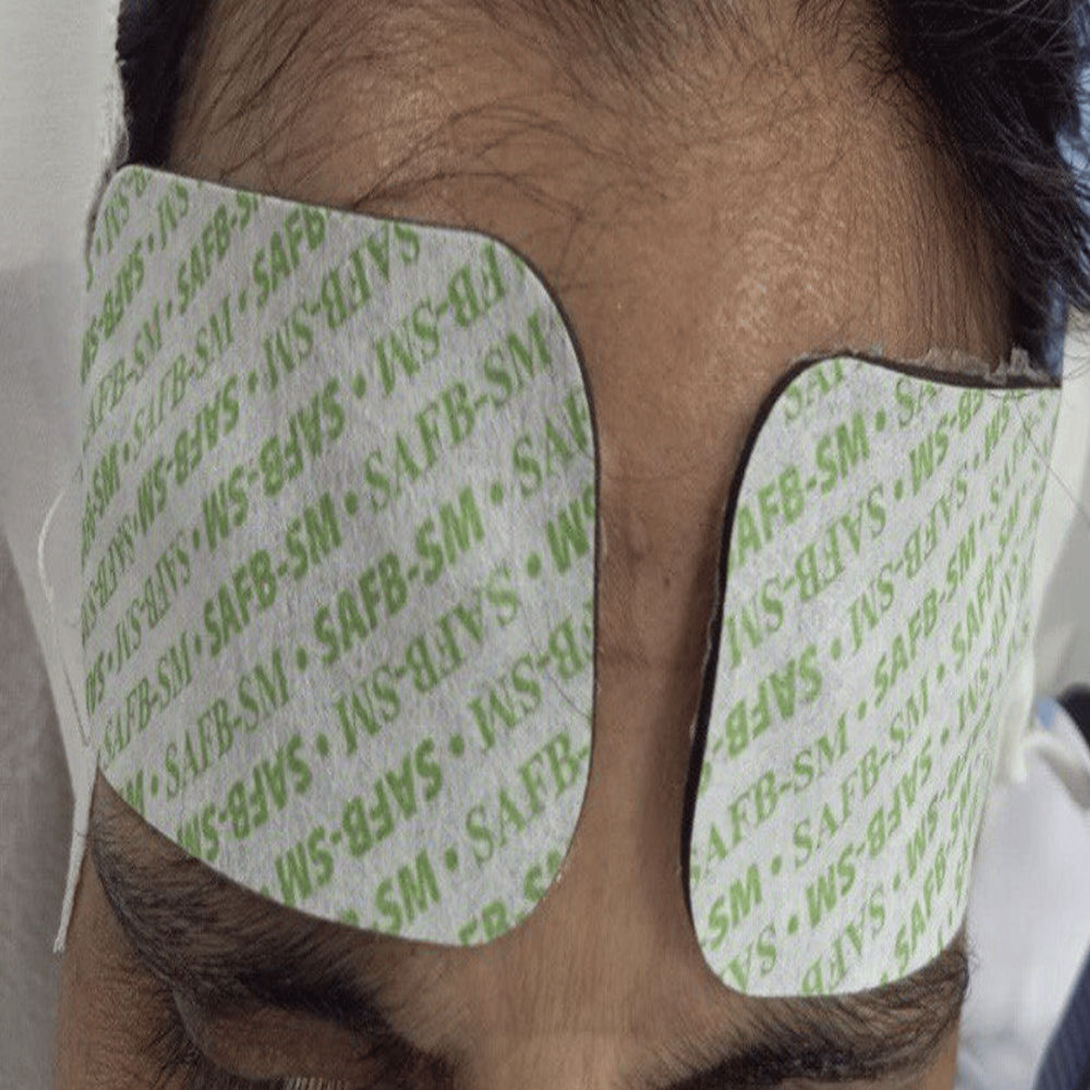
NIRS sensors attached to the forehead of a patient NIRS - near infrared spectroscopy

Using the NIRS machine, C-rSO₂ in the right and left frontal regions was noted continuously (Figure 2, Figure 3). Hypotension was considered a fall in MBP of more than 20% of the preoperative values. Patients who developed a fall in blood pressure (MBP fall of >20%) were treated with phenylephrine 50 to 100 microgram IV boluses or ephedrine 6 mg boluses as per the operating room anesthesiologist.

After the delivery of the neonate, APGAR scores were noted at one minute and five minutes post-delivery. Umbilical arterial blood samples were taken under sterile aseptic precautions and sent for arterial blood gas analysis. Postoperative delirium scores were calculated using a Delirium Rating Scale-98 (DRS-98) [8] on postoperative days one and two.

The statistical analysis was performed by SPSS version 23 (IBM Inc., Armonk, New York). Continuous variables were described as the mean and variation of each observation from the mean value (standard deviation), represented as mean ± SD (analyzed using the unpaired T-test) or median (IQR) (analyzed using the Mann-Whitney U test) if they did not follow a normal distribution. Categorical variables were described by taking percentages and analyzed using the chi-square test. Paired categorical data were analyzed using the McNemar test. Paired continuous data for three or more than three groups following a normal distribution were analyzed using the one-way ANOVA and Kruskal-Wallis test (if following a non-normal distribution).

Correlation analysis was done using the Pearson correlation test for parametric data and Spearman's correlation test for non-parametric data. Variables with a p-value <0.05 were considered statistically significant.

## Results

The incidence of hypotension in the first thirty minutes of spinal anesthesia in this study was sixty-four patients out of eighty-six (74.41%). In case of emergency cesarean, the incidence was 72.72% (16 out of 22 patients), and in case of elective cesarean, it was 75% (48 out of 64 patients). The incidence of neonatal outcomes was unaffected by maternal hypotension, with all neonates having Apgar scores of ≥7 at one and five minutes, and there were no significant differences in umbilical artery pH or PCO₂ levels between groups. Vasopressor analysis showed that ephedrine improved cerebral oxygenation more than phenylephrine. All the 22 emergency LSCS recruited in the study belonged to category three (NICE guideline (NG192)). Fifteen cases had previous LSCS in labor, four were breech presentations in labor, two had cephalopelvic disproportion, and one was LSCS on maternal request during labor.

The mean age of participants was 30.05 ± 4.72 years (Table 1). ⁣ The incidence of post-spinal hypotension was high, with an average MAP reduction of 40.33 ± 9.79 mmHg. Despite this, no significant correlation was seen between the fall in MAP and cerebral oxygen saturation, indicating cerebral autoregulation may compensate for changes in blood pressure (Table 2). The maximum fall in NIRS oxygenation occurred 1.03 minutes before the maximum fall in blood pressure, highlighting the sensitivity of NIRS in detecting early cerebral ischemia. The maximum fall in C-so2 was at a mean of 4.65 minutes, while the maximum fall in MBP occurred at a mean of 5.68 minutes.

**Table 1:** Demographics of the study population

Baseline variables	N=60
Age	30.05 ± 4.72
Weight	69.33 ± 11.87
Period of gestation	38.47 ± 1.34

**Table 2:** Correlation between the fall in MBP and the fall in rSO2 MBP - mean blood pressure; rSO2 - regional oxygenation; C-rSO2 - regional cerebral oxygenation

Correlation	p-value	R-value
Fall in MBP vs fall in left C-rSO2	0.058	0.246
Fall in MBP vs fall in right C-rSO2	0.955	-0.007
Fall in MBP vs fall in mean rSO2	0.835	-0.027
Time at maximal fall in MBP vs maximal fall in C-rSO2	0.001	0.412

Neonatal outcomes were unaffected by maternal hypotension, with all neonates having Apgar scores ≥7 at one and five minutes, and no significant differences in umbilical artery pH or PCO₂ levels between groups (Table 3). This indicates that autoregulation of uterine blood flow also remained intact. Vasopressor analysis showed that ephedrine improved cerebral oxygenation more than phenylephrine.

**Table 3:** Correlation between MBP fall and neonatal outcomes MBP - mean blood pressure

Correlation	p-value	R-value
Apgar 1 score vs fall in MBP	0.616	-0.066
Apgar 5 score vs fall in MBP	0.784	-0.036
Umbilical cord pH vs fall in MBP	0.059	0.245
Umbilical cord PCO2 vs fall in MBP	0.655	-0.059
Base excess vs fall in MBP	0.756	-0.041

No postoperative delirium was seen in any of the participants (Table 4).

**Table 4:** Postoperative delirium scores on POD1 and POD2 DrS-98 - Delirium Rating Scale-revised-98; POD - postopertative day

DRS-98 score	0	1	2	3	4	p-value
Day 1	18	24	12	5	2	0.013
Day 2	20	32	6	2	1	0.013

Vasopressor analysis showed that ephedrine improved cerebral oxygenation more than phenylephrine (Table 5). There is no significant effect of vasopressors on improving MBP and neonatal Apgar and acid-base status (Table 5).

**Table 5:** Vasopressor effects on MBP, C-rSO2 and neonatal outcomes MBP - mean blood pressure; C-rSO2 - regional cerebral oxygenation

Vitals	Ephedrine (mean ± SD)	Phenylephrine (mean ± SD)	Both (mean ± SD)	p-value
MP raise (%)	23.51 ± 10.64	25.68 ± 16.18	39.48 ± 11.77	0.096
RSO2 raise (%)	23.2 (12.47–31.66)	3.17 (0–6.74)	11.11 (6.22–32.87)	<0.001
Apgar score at 1 min	8.22 ± 1.21	8.43 ± 1.19	8.35 ± 1.17	0.794
Apgar at 5 minutes	9.5 ± 0.62	9.59 ± 0.69	9.4 ± 0.89	0.782
Umbilical cord pH	7.31 ± 0.06	7.31 ± 0.06	7.34 ± 0.05	0.426
Umbilical cord PCO2	39.2 ± 4.77	37.86 ± 2.87	37.32 ± 4.71	0.389

There was no significant correlation between fall in MBP and level of block achieved (Table 6).

**Table 6:** Correlation between the level of block achieved and fall in MBP MBP - mean blood pressure

Level of block achieved	T4	T5	T6	p-value
Fall in MBP (mean ±SD)	44.29 ± 14.68	37.29 ± 12.47	40 ± 5.65	0.62

## Discussion

Cerebral autoregulation is a key mechanism that keeps constant blood flow to the brain despite fluctuations in blood pressure. This autoregulatory range, between a MBP of 60 and 150 mm Hg, allows the brain to preserve perfusion despite changes in systemic pressures. However, tissue oxygen delivery is complex and depends on variables such as oxygen content, capillary density, cellular metabolism, intracerebral pressure, cerebral perfusion pressure, and central venous pressure, as this can significantly impair cerebral venous drainage when it is elevated, not just the MBP.

In this study, we observed that there was no significant correlation between the fall in MBP and the fall in C-rSO₂. This suggests that factors other than MBP may play a role in cerebral oxygenation. Our findings are consistent with a study conducted by MacEwen et al. [6], where no direct correlation was found between mean blood pressure and cerebral ischemia in patients undergoing hemodialysis.

Whereas the previous study by Fassoulaki et al. was conducted on thirty-four women undergoing elective cesarean delivery under spinal anesthesia. They noted a most remarkable decrease of C-rSO₂ in the right and left frontal regions five and 10 minutes after spinal injection. Similarly, Sun et al. conducted a study in patients undergoing elective cesarean section and found that the decrease in C-rSO₂ was high in parturients with spinal hypotension when compared to parturients without hypotension.

We also found C-rSO₂ fall was 1.03 minutes earlier than the fall in MBP, highlighting that C-rSO₂ can be a more sensitive marker of early ischemia than intermittent NIBP monitoring. Our findings are consistent with the study by Sun et al. [9], who found that C-rSO₂ changes could precede hypotension approximately 38 seconds earlier than intermittent blood pressure measurements. This time delay can be attributed to the method of intermittent non-invasive blood pressure measurement, which takes an average of 38.8 seconds, as per the study by Takahashi et al. [10]. The near-infrared spectroscopy (NIRS) device used for continuous oxygen monitoring provided near-real-time data on brain oxygen levels, making it a useful early warning tool for detecting ischemia compared to standard intermittent NIBP monitoring. During spinal anesthesia, the recognition of hypotension depends heavily on the frequency of the intermittent non-invasive blood pressure (NIBP) measurements. Typically, these measurements are assessed every three to five minutes; this may cause a significant delay in the detection of hypotensive episodes. Even a short duration of hypotension may adversely affect patient outcomes. Data from the NIRS device may be used in conjunction with continuous finger cuff non-invasive blood pressure measurement to diagnose and treat drops in C-rSO₂ and blood pressure in real time.

We found that the fall in MBP had no significant correlation with the level of block achieved after subarachnoid block. Though previous literature correlates the level of spinal anesthesia block and hypotension, they found that higher block levels are usually associated with a greater incidence of hypotension [11].

One study found that the time taken for the hypotension to occur was much shorter than the time to achieve maximum block height. A higher block may be associated with hypotension, but it does not always predict hypotension [12].

In a study conducted by Zhang et al. at a particular block level, some patients developed hypotension, and some of them did not develop hypotension. They found that instead of a particular block-level spinal block, the assertion rate is a good indicator for the prediction of hypotension [13].

In our study, we found that a fall in MBP had no significant correlation with the level of block achieved after subarachnoid block, as the block height achieved in all patients was between T4 and T6.

Previous studies have found that sustained post-spinal maternal hypotension results in neonatal acidosis [14]. In our study, there were no changes in neonatal Apgar scores at one and five minutes after spinal hypotension. There was no change in neonatal pH, pCO₂, and base excess after maternal spinal hypotension. This could be explained by the adequate use of vasopressors, preventing a sustained fall in blood pressure. This could also be explained by the fact that healthy neonates can tolerate brief periods of hypotension.

Another important finding of our study was that there was no significant increase in postoperative delirium scores in patients who experienced a post-spinal fall in cerebral oxygenation. This outcome is consistent with research by Harrison et al. [15], who similarly found no association between C-rSO₂ falls and postoperative delirium in patients over 60 years undergoing non-cardiac surgery. In contrast, studies on cardiac surgeries often show a link between cerebral oxygen desaturation and cognitive decline, prolonged hospital stay [16], and increased delirium incidence [76,^18^]. The differences in the incidence of postoperative delirium between cardiac and non-cardiac surgeries could be attributed to several factors. Cardiac surgery patients tend to be older, have more comorbidities [19], and experience additional surgical stressors [20], all of which increase their vulnerability to cognitive dysfunction. In our study, the lower incidence of postoperative delirium could be explained by the younger and healthier patient population (ASA 2) with a mean age of 30 years. Also, benzodiazepines were not used during this study. 

We found that the use of ephedrine (6 mg boluses) or a combination of ephedrine and phenylephrine (50 microgram boluses) resulted in a significant increase in rSO₂ values compared to phenylephrine alone (Table 4). However, there was no difference in the increase in MBP between the vasopressor groups.

Ephedrine is more effective in improving cerebral oxygenation because it acts as a mixed alpha- and beta-adrenergic agonist, thereby increasing cardiac output and improving cerebral blood flow. In contrast, phenylephrine, a selective alpha-adrenergic agonist, primarily causes vasoconstriction without significantly increasing cardiac output. Our findings are consistent with the studies that demonstrated phenylephrine use associated with a fall in C-rSO₂ [21,22]. However, when considering fetal outcomes, Cooper et al. [23] found that phenylephrine was associated with a lower risk of fetal acidosis compared to ephedrine, suggesting that phenylephrine may be preferable when considering fetal acid-base balance during cesarean deliveries. Although in our study, there was no significant impact on neonatal outcomes, as evidenced by stable Apgar scores and no significant changes in neonatal pH or PCO₂ values following maternal hypotension. These findings underscore that appropriate management of maternal hypotension with vasopressors, regardless of the agent used, can prevent adverse neonatal outcomes in healthy newborns. Also, this prompt vasopressor use maintained C-rSO₂ and potentially aborted any maternal adverse effects.

This study confirms that spinal block-induced hypotension is a common occurrence in cesarean deliveries but does not significantly affect cerebral oxygenation. NIRS provides early detection of cerebral ischemia and serves as a real-time monitor for managing maternal hemodynamic status. The lack of correlation between maternal hypotension and neonatal acid-base status supports that appropriate vasopressor treatment adequately maintains uteroplacental perfusion regardless of the type of vasopressor usage.

As this is an observational study, a major limitation of our study is the dose of local anesthetic given in the subarachnoid block, which was determined by the attending anesthesiologist. The dose given could have an impact on post-spinal hypotension. Other factors that influence post-spinal hypotension, like the BMI of the patient, height of the patient, etc., were also not taken into consideration. Transcutaneous NIRS measures only the first few millimeters of the frontal cortex, and thus, the rest of the cortical areas of the frontal cortex could not be analyzed. The fact that this study was observational in nature and the participants received intermittent NIBP monitoring is also a limitation of the study, as using invasive blood pressure monitoring or a non-invasive finger cuff monitor could have given real-time blood pressure monitoring.

Another major concern about the NIRS is undesired contamination of the C-rSO₂ by extracranial blood flow. Also, factors such as maternal anxiety causing tachycardia could have affected the cerebral oxygen values, and these factors were not analyzed in the study. Future multi-center studies with larger cohorts would be needed to validate the findings of this study.

## Conclusions

This study found that there was no significant fall in C-rSO₂ in relation to transient hypotension during spinal anesthesia. However, a fall in cerebral saturation occurred before the fall in blood pressure, rendering NIRS a potential real-time monitor if a continuous noninvasive blood pressure monitor is not available. Adverse neonatal outcomes due to hypotension can be prevented by avoiding prolonged hypotension through appropriate treatment with vasopressors. Both the ephedrine and phenylephrine were effective in maintaining blood pressure without any adverse neonatal outcomes, while the ephedrine has a role in improving maternal cerebral oxygenation compared to phenylephrine. The lack of correlation between maternal hypotension and neonatal acid-base status supports that appropriate vasopressor treatment adequately maintains uteroplacental perfusion regardless of the type of vasopressor used. 

## Figures and Tables

**Figure 4:**
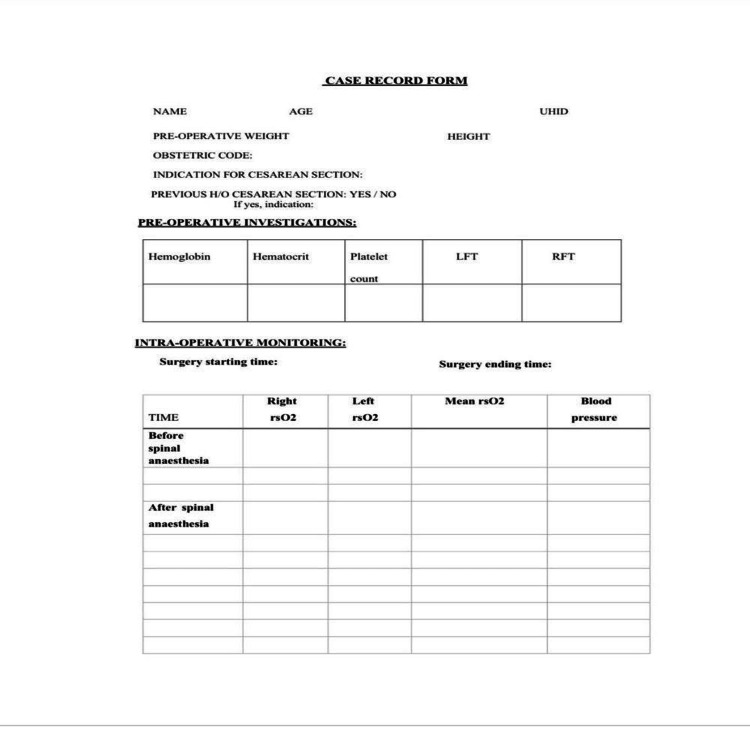
Case record

## Appendices

*Case record form*:

Case record form attached in Figure 4.
