# Correction: The Effect of Post-Spinal Hypotension on Cerebral Oxygenation Using Near Infrared Spectroscopy and Neonatal Outcomes in Full Term Parturients Undergoing Lower Segment Caesarean Section

**DOI:** 10.7759/cureus.c280

**Published:** 2025-08-29

**Authors:** Krithikabrindha Velusamy, Amit Kumar, Shailendra Kumar, Sana Y Hussain, Puneet Khanna, Nishant Patel

**Affiliations:** 1 Anesthesiology, All India Institute of Medical Sciences, Delhi, IND; 2 Anesthesiology, All India Institute of Medical Sciences, New Delhi, IND

This article has been corrected to remove the Delirium Rating Scale R-98 (DRS-R-98). The original version incorrectly included the scale in the Appendices without reprint permission. It was also incorrectly stated in the Appendix that the tool is freely available for academic and research purposes. However, it is under copyright.

Reference 8 has been corrected from:

Trzepacz PT: Delirium Rating Scale--Revised-98 (DRS-R-98). APA PsycTests. 1998, 10.1037/t28506-000

to the correct version:

Trzepacz PT,  Mittal D, Torres R, Kanary K, Norton J, Jimerson N: Validation of the Delirium Rating Scale-Revised-98: Comparison with the Delirium Rating Scale and the Cognitive Test for Delirium. J Neuropsychiatry Clin Neurosci. 2001, 10.1176/jnp.13.2.229

